# The impact of the scale and hierarchical structure of health human resources on the level of medical services-based on China’s four major economic regions

**DOI:** 10.1186/s12939-024-02239-8

**Published:** 2024-08-21

**Authors:** Jie-Ting Chen, Kai Yang, Yan Zhu, Xiang-Wei Wu

**Affiliations:** 1 The School of Medicine, Shihezi University, Shihezi, Xinjiang China; 2 The Academy of Education, Xinjiang Normal University, Urumqi, Xinjiang China

**Keywords:** Health Human resources, Medical Service, Four Major Economic regions, Panel Data, Fixed effects Model

## Abstract

**Background:**

Ensuring that the scale and hierarchical structure of health human resources are rational, and that medical services are efficient and fair, is an important task of practical significance. On this basis, examining the impact of health human resources on the level of medical services presents a new and formidable challenge. This study aims to delve into how the scale and hierarchical structure of health human resources in China’s four major economic regions affect the fairness and efficiency of medical services, and to identify optimization strategies.

**Methods:**

This study utilizes provincial panel data from China’s four major economic regions spanning the years 2009 to 2021. Initially, it provides a statistical description of the current state of health human resources and the level of medical services. Subsequently, it employs a fixed-effects model to analyze the impact of the scale and hierarchical structure of health human resources, as well as their interactive effects, on the fairness and efficiency of medical services, and discusses the interactive mechanisms between medical service fairness and medical service efficiency. Furthermore, after conducting a comprehensive evaluation of the level of medical services using the entropy weight method, it explores the regional heterogeneity and temporal dynamics in the influence of the scale and hierarchical structure of health human resources on the level of medical services. Finally, the study examines the scientific validity and rationality of the research findings through various robustness checks, including the substitution of research variables and models.

**Results:**

The study found that the scale of health human resources has a promoting effect on the equity of medical services (β ≤ 0.643, *p* ≤ 0.01), but exhibits an inhibitory effect on the efficiency of medical services (β ≥ -0.079, *p* ≤ 0.1); the hierarchical structure of health human resources shows a positive impact on both the equity and efficiency of medical services (β_equity_ ≤ 0.160, *p* ≤ 0.01; β_efficiency_ ≤ 0.341, *p* ≤ 0.05); at the same time, the results indicate that the interactive effect of the scale and hierarchical structure of health human resources promotes equity in medical services (β = 0.067, *p* ≤ 0.01), but restricts the efficiency of medical services (β ≥ -0.039, *p* ≤ 0.01); the mechanism by which health human resources affect the level of medical services in China’s western and northeastern regions is more pronounced than in the central and eastern regions; after the implementation of the “Healthy China 2030” Planning Outline, the role of health human resources in the level of medical services has been strengthened; in the robustness tests, the model remains robust after replacing the core explanatory variables, with R^2^ maintained between 0.869 and 0.972, and the dynamic GMM model test shows a significant second-order lag in the level of medical services (β_equity_ ≤ 0.149, *p* ≤ 0.01; β_efficiency_ ≤ 0.461, *p* ≤ 0.01); the channel test results prove that managerial personnel and other technical personnel are key pathways in regulating the impact of medical staff on the level of medical services.

**Conclusion:**

This study provides an in-depth analysis of the impact of health human resources on the level of medical services, revealing that both the scale and hierarchical structure of health human resources significantly affect the equity and efficiency of medical services. Furthermore, the influence of health human resources on the level of medical services exhibits regional heterogeneity and temporal characteristics. Robustness tests ensure the scientific validity and robustness of the research conclusions. This provides effective references for optimizing the allocation of health human resources and improving the level of medical services.

## Background

Health human resources, as the key to implementing health plans and building a healthy China, form the foundation of the supply of medical services. Medical services play a crucial role in maintaining and restoring health, extending life cycles, and improving the quality of life. The United Nations General Assembly called for universal healthcare coverage as a primary goal at its seventieth session [1]. However, the uneven distribution of health human resources and the insufficiency of medical service supply remain global issues [2, 3]. Therefore, the World Health Organization (WHO) emphasizes the need to achieve sustainable development of global health human resources and effectively support universal health coverage and improve global health standards [4].

Since the reform and opening up, China has gradually implemented healthcare system reforms, significantly improving the coverage and level of basic medical insurance. However, with the transformation of the socio-economic landscape, medical resources have gradually become insufficient, leading to new challenges for the medical service system. In response, after implementing the “Deepening of Medical and Health System Reform” in 2009 (also known as the “New Medical Reform” in China), China launched the “Tiered Diagnosis and Treatment” system and further proposed in 2014 to “Strengthen the Capacity Building of Primary-level Medical and Health Services.”

With the aging population, changes in the disease spectrum, and shifts in the ecological environment and lifestyle, systems such as the “New Medical Reform” are beginning to show fatigue in addressing the mismatch between the supply and demand of health and wellness services. At the same time, due to the overall shortage of medical resources, the unreasonable hierarchical structure, the imbalance of medical service supply and demand, and the large regional distribution differences in China, people have started to seek higher quality medical services through out-of-place medical treatment [5–7]. Although the government began to implement the “Healthy China 2030” Planning Outline in 2016 (referred to as the “Outline”) to alleviate issues such as “difficulty and high cost of seeing a doctor,” by 2022, the total number of health personnel was only 14 million, with a doctor-nurse ratio of 1:1.17, and issues such as the scarcity of health resources in the western region and the overflow of health resources in the eastern region still exist. Therefore, the National Health Commission issued the “14th Five-Year Health and Health Standardization Work Plan” and the “14th Five-Year Health and Health Talent Development Plan” in 2022, calling for further optimization of health human resources and improvement of service capabilities, encouraging regions to promote a balanced layout of high-quality medical resources and to improve the homogenization level of medical services in different areas.

In recent years, the development status and influencing factors of health human resources allocation have become the main focus of scholars. Research is mainly divided into two directions: on the one hand, scholars study the quantity, proportion, and growth rate of health human resources allocation from a macro perspective, striving to grasp the overall situation of health human resources [8–11]. Vedanthan found that there is a significant difference in the global distribution of health human resources, with developed countries often having more medical resources, while developing countries, especially in rural or remote areas, generally suffer from a shortage of health human resources. In recent years, influenced by economic recession, some developed countries also face a shortage of health human resources [2, 12]. On the other hand, scholars analyze the impact of geography, economy, population, education, and technology on health human resources from a meso or micro perspective, and propose management and development plans accordingly [13–17]. Pálsdóttir and Uwizeye and others found after in-depth research that optimizing the allocation of health human resources is considered to significantly accelerate the overall development process of society and economy [18, 19]. In addition, health human resources, as direct providers of medical services, are considered by some scholars to have a very close connection with them [20]. On this basis, influenced by utilitarian ethics and the theory of equal distribution, among other theoretical frameworks, researchers further focus on the two core dimensions of medical service efficiency and medical service equity [21, 22]. In terms of medical service efficiency, scholars focus more on how to maximize medical service efficiency under limited resource conditions [23–25]. Researchers not only analyze various factors affecting medical service efficiency but also try to introduce new medical models such as telemedicine and smart healthcare to improve the efficiency of medical service supply [26–28]. Herwartz and Xian-Hui He and others have revealed the impact mechanism of socio-economic factors on medical resources by evaluating the efficiency of medical and health institutions [29, 30]. Jessup and others have also attempted to find alternative models to improve medical service efficiency [31]. At the same time, scholars pay more attention to the equity and accessibility of medical services and propose a series of policy recommendations to promote medical service equity [32, 33]. For example, Mongolian scholar Erdenee conducted comparative studies between urban and rural areas through the Mann-Whitney U test, using the Gini coefficient to further investigate the distribution equity of health resource allocation [34]. Chinese scholars such as Zhang Yue and others have conducted in-depth analysis of the equity of primary healthcare resource allocation in mainland China using tools such as the Lorenz curve, Gini coefficient, Theil index, and health resource density index [35].

Generally speaking, most studies are limited to a single dimension and have not systematically revealed the multidimensional mechanisms of the impact of health human resources on the level of medical services; moreover, previous studies have mostly divided the research area into eastern, central, and western regions, with fewer studies on the four major economic regions; at the same time, the methods chosen by scholars for robustness tests are relatively singular, which may lead to a more one-sided understanding. Therefore, this study, by taking the four major economic regions of China as the research subjects, analyzes the impact of the scale and hierarchical structure of health human resources on the fairness and efficiency of medical services, using a variety of test methods to conduct a comprehensive and rigorous robustness test of the research results, providing a reference for the planning of health human resources and the improvement of the level of medical services in China.

## Methods

### Data source

The original data for this study were compiled from official statistical yearbooks published in various years. Specifically, the number of licensed (assistant) physicians, registered nurses, the number of hospitals per 10,000 square kilometers, the number of hospital beds per thousand people, the number of outpatient services enjoyed per capita, working days of hospital beds, average length of hospital stay, the number of discharges per bed, government health expenditure, social health expenditure, and personal health expenditure were derived from the “China Health Statistics Yearbook” from 2010 to 2012, the “China Health and Family Planning Statistics Yearbook” from 2013 to 2017, and the “China Health and Health Statistics Yearbook” from 2018 to 2022. The per capita GDP and the total population at the end of the year came from the “China Statistical Yearbook” of each corresponding year (see Table 1). Considering that there was some missing data in 2009, and the Grey Prediction Model is characterized by requiring less data and having high predictive accuracy, this study chose this model to complete the missing data [36, 37].

**Table 1:** Indicator system and measurement

Variable Classification	Variable Meaning	Calculation Method or Source	Indicator Nature	Unit
Dependent Variable	Medical Service Equity	Number of hospitals per 10,000 square kilometers	+	/
		Number of hospital beds per thousand people	+	/
		Number of hospital outpatient services per capita	+	Time
	Medical Service Efficiency	Hospital bed working days	+	Day
		Average hospital stay	+	Day
		Number of discharged patients per bed	+	person
Core Explanatory Variables	Scale of Health Human Resources	Total number of practicing assistant physicians and registered nurses	+	person
	Hierarchical Structure of Health Human Resources	Ratio of practicing assistant physicians to registered nurses	-	/
Control Variables	Total population at the year-end	From statistical yearbooks	+	10,000 persons
	Government health expenditure	From statistical yearbooks	+	100 million CNY
	Personal health expenditure	From statistical yearbooks	+	100 million CNY
	Social health expenditure	From statistical yearbooks	+	%
	GDP	From statistical yearbooks	+	100 million CNY

CNY: Chinese yuan; GDP: Gross Domestic Product

### China’s four major economic regions

**Fig. 1:**
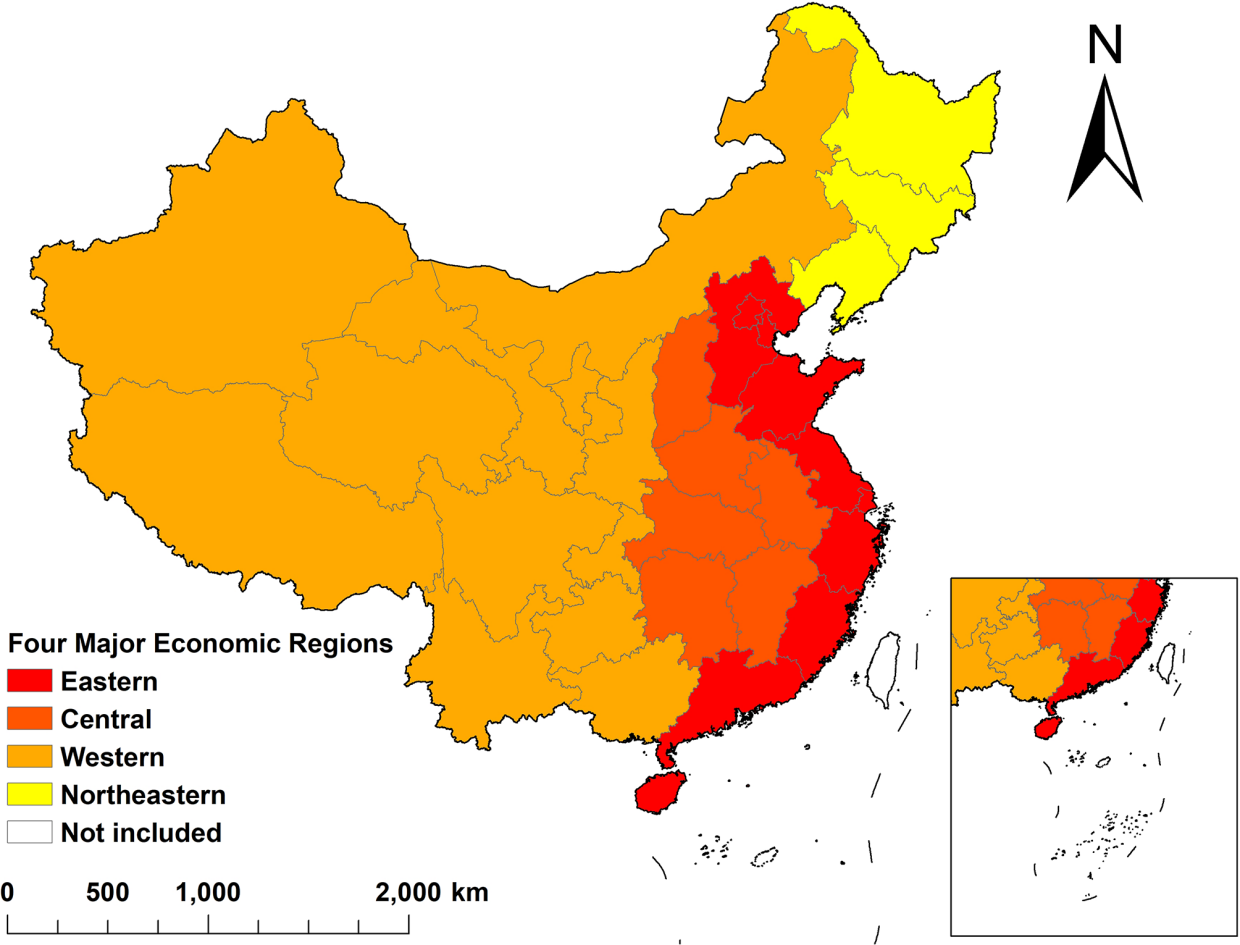
The four major economic regions of China

In accordance with the “Several Opinions on Promoting the Rise of the Central Region” by the CPC Central Committee and the State Council, the “Implementation Opinions on Several Policy Measures for the Development of the Western Region” issued by the State Council, and the spirit of the report at the 16th National Congress of the Party, China is divided into four major economic regions (see Fig. 1). The Northeast region includes: Liaoning, Jilin, Heilongjiang; the Eastern region includes: Beijing, Tianjin, Hebei, Shanghai, Jiangsu, Zhejiang, Fujian, Shandong, Guangdong, Hainan, Taiwan, Hong Kong, Macao; the Central region includes: Shanxi, Anhui, Jiangxi, Henan, Hubei, Hunan; the Western region includes: Inner Mongolia, Guangxi, Chongqing, Sichuan, Guizhou, Yunnan, Tibet, Shaanxi, Gansu, Qinghai, Ningxia, Xinjiang. Since the relevant research variables in the Health Statistics Yearbook do not cover Taiwan, Macao, and Hong Kong, these three regions are excluded from this study. Considering the unique socio-economic and developmental conditions of different regions in China, this study takes China’s four major economic regions as the research subjects [38].

### Health personnel

Health personnel refer to professionals working in medical and health institutions. According to the China Health Statistics Yearbook, health personnel in hospitals at all levels in China are categorized into four groups: medical and health technicians, other technicians, administrative managers, and support staff. Medical and health technicians include licensed physicians, assistant physicians, registered nurses, pharmacists, laboratory technicians, imaging technicians, health inspectors, and trainee medical (pharmacy, nursing, technical) practitioners and other health professionals. Other technicians are non-health professionals engaged in technical work such as medical equipment maintenance, health promotion, scientific research, and teaching. Administrative managers are staff members who undertake leadership responsibilities or administrative tasks. Support staff are those who perform skilled operations and maintenance, logistical support services, and other related duties, including nursing assistants, pharmacy assistants, laboratory assistants, fee collectors, registration clerks, etc. Licensed (assistant) physicians and registered nurses constitute the majority of health personnel and are the main force in providing medical services; therefore, this study considers licensed (assistant) physicians and registered nurses as the core explanatory variables.

### Fixed effects model

This study selects the fixed effects model to analyze the impact of the scale and hierarchical structure of health human resources on the level of medical services. According to the specific model setup, it can be further divided into the static fixed effects model and the dynamic panel regression model. The static fixed effects model is relatively simple, assuming that each individual’s regression coefficient is fixed and does not change over time, which can effectively handle the variance and heterogeneity issues of the dependent variable, and can be used for both single-variable and multi-variable analysis. The dynamic panel regression model can consider the time effect, expanding the model to a dynamic model [39]. Considering that the research data is a mixed data containing time series and cross-sectional data, a panel regression model is chosen [40]. The specific formula can be implemented as follows:

1 $$\:{ln}\left({y}_{it}\right)={a}_{i}+\varnothing\:{ln}({y}_{it}-1)+\beta\:{ln}{(x}_{it})+{u}_{it}$$

In which, $\:{y}_{it}$ represents the level of medical services, $\:x$ represents the hierarchical structure of health human resources, $\:t$ represents the year, $\:i$ represents the province, $\:\varnothing\:$ represents the coefficient of the corresponding autoregressive explanatory variable, $\:\beta\:$ represents the coefficient of the corresponding exogenous explanatory variable, $\:a$ is the intercept term, and $\:u$ is the random error term.

### Entropy weight method

The Entropy Weight Method is an evaluation approach that determines the weights of various assessment indicators based on the concept of information entropy, used to quantify the degree of dispersion among indicators. This method can eliminate the impact of different dimensions when the indicators have significantly different units of measurement [41, 42]. Considering the indicators used in this study are not dimensionally homogeneous, the Entropy Weight Method is adopted to conduct a comprehensive evaluation of the medical service level across China’s four major economic regions. The specific implementation steps are as follows:

#### Standardization treatment

First, each indicator is dimensionless. For positive indicators, an ascending gradient fuzzy membership function is used; for negative indicators, a descending gradient fuzzy membership function is used.

2 $$\:{y}_{ij}=\left\{\begin{array}{c}\frac{{x}_{ij}-min\left({X}_{j}\right)}{max\left({X}_{j}\right)-min\left({X}_{j}\right)},{X}_{j}\:represents\:a\:positive\:indicator\\\:\frac{max\left({X}_{j}\right)-{x}_{ij}}{max\left({X}_{j}\right)-min\left({X}_{j}\right)},{X}_{j}\:represents\:a\:negative\:indicator\end{array}\right.$$

In which $\:{x}_{\text{i}\text{j}}$ is the actual value of the *j*th indicator for the *i*th province, $\:{y}_{ij}$ is its degree of membership in the fuzzy set; $\:min\left({X}_{j}\right)$ and $\:max\left({X}_{j}\right)$ are the minimum and maximum values of the *j*th indicator, respectively.

#### Calculate the weight of the *i*th province’s *j*th indicator *p*

Calculate the proportion of the *i*th sample in the *j*th indicator, which is the weight value of the *i*th sample in the *j*th indicator.

3 $$\:{p}_{ij}=\frac{{y}_{ij}}{{\sum\:}_{i}^{n}{y}_{ij}},$$

In which $i=\text{1,2},\ldots,n$, and $j=\text{1,2},\ldots,m$.

#### Calculate the information entropy *E*

Calculate the entropy value of a certain indicator.

4 $$\:{E}_{j}=-\frac{1}{ln\left(n\right)}\sum\:_{i=1}^{n}{p}_{ij}ln\left({p}_{ij}\right)$$

In which, *n* represents the sample size, and generally speaking, $\:0\le\:{E}_{j}\le\:1$.

#### Determine the weight of each indicator *w*

Based on the calculation formula of information entropy, calculate the entropy of each indicator, and then calculate the weight of each indicator through the entropy.

5 $$\:{W}_{j}=\frac{1-{E}_{j}}{m-\sum\:{E}_{j}}\:(j=\text{1,2},\ldots,m)$$

#### Calculate the comprehensive score

6 $$\:{Score}_{ij}=\sum\:_{j=1}^{m}{W}_{j}\bullet\:{y}_{ij}\:(i=\text{1,2},\ldots,n)$$

### Data analysis

This study employs descriptive statistical methods to elucidate the current state of health human resources and the level of medical services. Utilizing StataMP 16 software, a fixed effects model is constructed to analyze the impact of the scale and hierarchical structure of health human resources on the equity and efficiency of medical services. Additionally, robustness checks are performed through regional heterogeneity tests, temporal stage examinations, substitution of core explanatory variables, application of the dynamic Generalized Method of Moments (GMM) model, and channel tests.

## Results

### Development status of health human resources and medical service level

This study utilizes the provincial panel data of China’s four major economic regions spanning the years 2009 to 2021, and a descriptive statistical analysis of the current situation of medical service level and health human resource allocation is conducted, as detailed in Table 2. There is a large difference in the number of hospitals per 10,000 square kilometers, personal health expenditure, and regional GDP, which may be the main factors affecting the level of medical services, while indicators such as hospital bed working days, the number of discharges per bed, and the ratio of medical staff to nursing staff show smaller differences.

**Table 2:** Descriptive statistics of variables

Variable Meaning	Symbol	Sample Size	Mean	Standard Deviation	Minimum Value	Maximum Value
Number of hospitals per 10,000 square kilometers	lnh	403	3.777	1.354	-0.206	6.516
Number of hospital beds per thousand people	lnhb	403	1.330	0.315	0.500	2.718
Number of hospital outpatient services per capita	lnhs	403	0.766	0.453	-0.300	2.056
Hospital bed working days	lnbwd	403	5.707	0.106	5.172	5.902
Average hospital stay	lnals	403	2.431	0.535	2.092	4.445
Number of discharges per bed	lndpb	403	3.356	0.152	2.724	3.880
Total number of practicing (assistant) physicians and registered nurses	lnshr	403	11.511	0.897	8.189	13.031
practicing (assistant) physicians to registered nurses	lnhhr	403	-0.288	0.184	-0.571	0.618
Total population at the year-end	lnp	403	10.423	0.843	7.996	11.751
Government health expenditure	lnghe	403	5.714	0.783	3.057	7.576
Personal health expenditure	lnphe	403	5.650	1.057	1.166	7.512
Social health expenditure	lnshe	403	3.599	0.235	2.904	4.134
Regional GDP	lngdp	403	9.669	1.020	6.090	11.731

### Impact of the hierarchical structure of health human resources on medical service equity

This study selects the fixed effects model based on the results of the Hausman test and constructs the following models for analysis: Model (1) takes the “number of hospitals per 10,000 square kilometers” as the dependent variable to test the impact of health human resources on the equity of medical services from various perspectives; Model (3) takes the “number of hospital beds per 1,000 people” as the dependent variable; Model (5) takes the “number of hospital outpatient services per capita” as the independent variable. Models (2), (4), and (6) further introduce the interaction terms between the scale and hierarchical structure of health human resources based on (1), (3), and (5), respectively. The results are presented in Table 3.

In Table 3, the impact of different dependent variables on the degree of medical service equity increases in the following order: “number of hospitals per 10,000 square kilometers > number of hospital beds per 1,000 people > number of outpatient services per capita.” Columns (1) and (2) show that both the scale and hierarchical structure coefficients are significant and positive, indicating that the allocation of medical and nursing personnel has a positive effect on the equity of medical resources. The interaction term is also significantly positive at the 1% level, and government health expenditure and regional gross domestic product significantly affect, indicating that traditional economic factors are the main reasons for the equalization of regional medical resources. However, under the regulation of the scale of health human resources, it will have a positive impact on the equity of medical services. But through columns (3) and (4), it can be found that although the scale and hierarchical structure have a positive effect on the number of outpatient services per capita, after the introduction of the interaction term, the significance of both the interaction term and the hierarchical structure has changed simultaneously, indicating that the scale of doctors and nurses has a negative regulatory effect on the impact of the medical-nurse ratio on the equity of medical services. This indirectly proves the need to shift from increasing quantity to improving quality to promote the equity of medical services. The significant negative effect of the total population also confirms this view. The results of columns (5) and (6) are basically consistent, with social health expenditure showing a significant positive effect in the control variables, while the regional gross domestic product is significant and the coefficient has increased slightly, indicating that the scale of health human resources is still the core element supporting the output of medical services. As the number of medical services per capita increases, it will more rely on factors such as the scale of health human resources and regional economic and social investment. This phenomenon is closely related to the current widespread demand of the people, that is, to meet the growing demand for primary medical services by expanding health human resources and injecting a large number of “basic human capital” into medical and health institutions.

**Table 3:** The impact of health human resource scale and hierarchical structure on medical service equity

	(1)	(2)	(3)	(4)	(5)	(6)
	lnh		lnhb		lnhs	
lnshr	1.074 ***	1.062***	0.853***	0.851***	0.643***	0.646***
	(15.720)	(15.660)	(18.280)	(18.180)	(11.100)	(11.140)
lnhhr	0.341***	1.039***	0.160***	0.265	0.015	-0.210
	(4.560)	(3.850)	(3.130)	(1.420)	(0.230)	(-0.910)
lnshr*lnhhr		0.067***		-0.010		0.021
		(-2.690)		(-0.590)		(1.010)
lnp	0.038	0.067	-1.040***	-1.035***	-0.919***	-0.928***
	(0.690)	(1.210)	(-27.730)	(-27.040)	(-19.760)	(-19.570)
lnghe	-0.087**	0.073*	0.046	0.048	-0.049	-0.053
	(-1.990)	(-1.670)	(1.540)	(1.600)	(-1.320)	(-1.430)
lnphe	0.010	-0.009	-0.026	-0.026	-0.010	-0.010
	(-0.30)	(-0.270)	(-1.100)	(-1.090)	(-0.340)	(-0.350)
lnshe	0.026	-0.008	0.040	0.035	0.146***	0.156***
	(0.480)	(-0.150)	(1.090)	(0.930)	(3.220)	(3.360)
lngdp	-0.132***	-0.136***	0.055**	0.054**	0.105***	0.106***
	(-3.960)	(-4.120)	(2.420)	(2.390)	(3.710)	(3.760)
Intercept	Yes	Yes	Yes	Yes	Yes	Yes
Observations	403(31)	403(31)	403(31)	403(31)	403(31)	403(31)
R^2^	0.998	0.998	0.984	0.984	0.988	0.988

The t values are in parentheses below the coefficients; *, *, and *** represent significance at the 0.1, 0.05, and 0.01 levels, respectively

### Impact of the hierarchical structure of health human resources on medical service equity

On the premise of ensuring the fairness and stability of medical services, the process of improving the efficiency of medical services and achieving health promotion is jointly influenced by economic, demographic, and social factors. The quality of personnel required to ensure medical service fairness and to enhance the efficiency of medical services varies, hence the effects of health human resources also differ. This section constructs the following models for analysis: Models (1), (3), and (5) respectively examine the impact of health human resources on “hospital bed workdays,” “average length of hospital stay,” and “discharges per bed”; Models (2), (4), and (6) further introduce interaction terms based on the previous models. The results are presented in Table 4.

In Table 4, only columns (1) and (2) indicate that the scale of health human resources is positively significant, suggesting that the expansion of the scale of health human resources can to some extent promote the improvement of medical service efficiency. However, to further optimize medical service efficiency, it is necessary to start from the hierarchical structure, which is consistent to some extent with the research of scholars such as Nyawira [43]. Compared with columns (1) and (3), after introducing the interaction terms of scale and hierarchical structure in columns (2) and (4), the hierarchical structure of health human resources changes from insignificant to significant, and the interaction terms are significantly negative, which also indirectly indicates that the hierarchical structure of health human resources needs to be at an appropriate scale to promote the improvement of medical service efficiency. Overexpansion of the scale of health human resources may instead restrict the positive role of the hierarchical structure in medical services. In columns (5) and (6), the coefficients of scale, hierarchical structure, and interaction terms are all insignificant, which may be due to the saturation of hospital service capacity, keeping the number of discharges per bed at a certain level, unaffected by health human resources. In addition, among most control variables, only the regional gross domestic product has a significant positive effect, indicating that the promoting effect of the scale and hierarchical structure of health human resources on the number of discharges per bed is not significant, but rather the level of regional economic development has become the main driving factor for people to be hospitalized for treatment. The effects of social and government health inputs are unstable, which may be related to multicollinearity between variables.

**Table 4:** The impact of health human resource scale and hierarchical structure on medical service efficiency

	(1)	(2)	(3)	(4)	(5)	(6)
	lnbwd		lnals		lndpb	
lnshr	-0.079*	-0.086**	0.050	0.022	-0.067	-0.063
	(-1.970)	(-2.150)	(0.780)	(0.370)	(-1.070)	(-1.010)
lnhhr	-0.069	0.341**	-0.004	1.673***	-0.017	-0.253
	(-1.570)	(2.140)	(-0.060)	(7.020)	(-0.250)	(-1.010)
lnshr*lnhhr		-0.039***		-0.161***		0.023
		(-2.670)		(-7.320)		(0.980)
lnp	0.010	0.027	-0.010	0.061	0.040	0.030
	(0.310)	(0.830)	(-0.190)	(1.240)	(0.790)	(0.580)
lnghe	0.006	0.015	0.041	0.075*	-0.041	-0.045
	(0.250)	(0.560)	(1.00)	(1.940)	(-1.010)	(-1.120)
lnphe	-0.032	-0.032	-0.005	-0.002	-0.018	-0.018
	(-1.580)	(-1.560)	(-0.150)	(-0.070)	(-0.550)	(-0.570)
lnshe	0.013	-0.007	0.117**	0.036	-0.043	-0.032
	(0.410)	(-0.220)	(2.350)	(0.750)	(-0.880)	(-0.630)
lngdp	0.078***	0.076***	0.099***	0.088***	0.084***	0.085***
	(3.980)	(3.870)	(3.180)	(3.030)	(2.730)	(2.780)
Intercept	Yes	Yes	Yes	Yes	Yes	Yes
Observations	403(31)	403(31)	403(31)	403(31)	403(31)	403(31)
R^2^	0.893	0.893	0.989	0.989	0.873	0.873

The t values are in parentheses below the coefficients; *, *, and *** represent significance at the 0.1, 0.05, and 0.01 levels, respectively

### Interactive mechanism between medical service fairness and efficiency

Fair and reasonable allocation of medical services is the foundation for high-efficiency medical service output, and efficient medical services can, in turn, promote medical service fairness. To independently examine the effects of human health resources, the interactive mechanism needs to be removed. Due to space limitations, only two representative variables, “number of hospital beds per thousand people” and “average length of hospital stay,” are selected to control the impact of service fairness on efficiency and the feedback effect of service efficiency on fairness in each other’s models (the results are shown in Table 5).

In Table 5, under the consistent conditions of health human resource scale and hierarchical structure and interactivity, both medical service efficiency and fairness are significantly positive at the 1% level, indicating that there is a positive interactive mechanism between the two types of activities. The other results are consistent with the previous text, and the conclusions are robust.

**Table 5:** The interactive mechanism between medical service fairness and efficiency

	(1)	(2)	(3)	(4)
	lnhb		lnals	
lnshr	0.751***	0.753***	0.096*	0.118**
	(11.880)	(12.050)	(1.660)	(2.130)
lnhhr	-0.138	0.826**	0.079	1.472***
	(-1.630)	(2.590)	(1.180)	(6.450)
lnshr*lnhhr		-0.09***		0.132***
		(-3.130)		(-6.350)
lnals	0.233***	0.157**		
	(3.490)	(2.230)		
lnhb			0.142***	0.088**
			(3.490)	(2.230)
Intercept	Yes	Yes	Yes	Yes
Observations	403(31)	403(31)	403(31)	403(31)
R^2^	0.949	0.949	0.989	0.989

The t values are in parentheses below the coefficients; *, *, and *** represent significance at the 0.1, 0.05, and 0.01 levels, respectively

### Regional heterogeneity test based on the four major economic regions

This study selects provincial panel data from China’s eastern, central, western, and northeastern economic regions as subjects for testing regional heterogeneity. The reason is that external conditions such as regional economic development and policy support are the main factors restricting the development of the medical and health industry. The four major economic regions of China differ in economic development and policy support. Using the four major economic regions of China as research subjects can not only reflect common issues from a global perspective but also fully consider individual development situations. At the same time, it also excludes the possibility that the effect of the interaction between the scale and hierarchical structure of health human resources may be due to regional heterogeneity. According to the division of the four major economic regions in relevant documents, and considering the availability of data, regional heterogeneity tests are carried out in 31 provinces, municipalities, and autonomous regions of China, excluding three provinces of Taiwan, Hong Kong, and Macao (see Table 6).

In Table 6, the scale of health human resources is significantly positive for the level of medical services in each column, consistent with the results of the previous text. However, after observing the coefficients of the hierarchical structure and interaction terms, it can be found that only the western and northeastern regions are significant, while the eastern and central regions are not significant. On the one hand, this indicates that the eastern and central regions, due to their advantageous geographical location and leading economic development, have attracted a large number of high-quality talents. This has led to an overflow of health human resources in first-tier cities, resulting in an established pattern of health human resources structure imbalance, hence the role of this hierarchical structure in the level of medical services is not significant. On the other hand, the western region is suffering from a talent shortage, and the northeastern region is experiencing talent loss, leading to a lack of coordination between the scale and hierarchical structure of health human resources in both regions, resulting in a significantly negative interaction term coefficient. In general, among China’s four major economic regions, the scale and hierarchical structure of health human resources show regional heterogeneity in their impact on the level of medical services. The lack of coordination between the scale and hierarchical structure of health human resources has, to some extent, constrained the improvement of medical service capabilities.

**Table 6:** Regional heterogeneity of health human resource scale and hierarchical structure on medical service level

	(1)	(2)	(3)	(4)	(5)	(6)	(7)	(8)	(9)	(10)
	Overall		East		Central		West		Northeast	
lnshr	0.747***	0.714***	1.081***	1.133***	0.780***	0.885***	0.841***	0.790***	1.119***	0.822***
	(7.920)	(7.800)	(3.370)	(3.300)	(5.650)	(2.960)	(5.220)	(5.190)	(4.800)	(3.170)
lnhhr	-0.105	2.060***	-0.771	-1.887	0.091	-1.198	0.409**	2.096***	-0.589***	5.284*
	(-0.800)	(4.590)	(-1.560)	(-0.710)	(0.530)	(-0.370)	(2.030)	(4.690)	(-3.610)	(1.880)
lnshr*lnhhr		-0.204***		0.093		0.107		-0.169***		-0.547**
		(-5.020)		(0.430)		(0.400)		(-4.170)		(-2.100)
Intercept	Yes	Yes	Yes	Yes	Yes	Yes	Yes	Yes	Yes	Yes
Observations	403(31)	403(31)	130(10)	130(10)	78(6)	78(6)	156(12)	156(12)	39(3)	39(3)
R^2^	0.974	0.976	0.976	0.976	0.995	0.995	0.976	0.979	0.998	0.998

The t values are in parentheses below the coefficients; *, *, and *** represent significance at the 0.1, 0.05, and 0.01 levels, respectively

### Time heterogeneity test based on the “healthy China 2030” plan

This study selects provincial panel data from China’s eastern, central, western, and northeastern economic regions for testing regional heterogeneity. The CPC Central Committee and the State Council issued and implemented the “Healthy China 2030” Planning Outline (referred to as the “Outline”) on October 25, 2016. To examine whether this programmatic policy has intervened in the impact of the scale and hierarchical structure of health human resources on the level of medical services, 2016 was chosen as the temporal breakpoint. Two sets of panel data, 2009–2016 and 2017–2021, were used to replicate the empirical tests mentioned above. Considering that from the end of 2019 to the end of the observation period, the country was affected by the Corona Virus Disease 2019 (COVID-19), to eliminate the interference of this event on the test of temporal heterogeneity, 2019 was supplemented as the second temporal breakpoint. The test results are presented in Table 7.

From Table 7, it can be seen that before the implementation of the Outline, the stock of health human resources was still the main driving force for improving the level of medical services. The number of doctors and nurses was the main regulatory channel for the hierarchical structure. The promotional effect of health human resources on the level of medical services was mainly concentrated in 2017–2019. The empirical test confirmed that policy intervention has a very prominent positive effect on the rational allocation of health human resources and the improvement of the level of medical services. Comparing and analyzing the results of 2017–2019 with those of 2020–2021, it can be found that COVID-19, as a sudden public health event, has a significant inhibitory effect on the provision of medical services by health human resources. The significant increase in the coefficient of the scale of health human resources may be related to the expansion of medical staff during the pandemic.

**Table 7:** Temporal heterogeneity of health human resource scale and hierarchical structure on medical service level

	(1)	(2)	(3)	(4)	(5)	(6)
	2009-2016year		2017-2019year		2020-2021year	
lnshr	0.416***	0.394***	0.765***	0.785***	-6.017**	-5.993**
	(7.900)	(7.580)	(5.080)	(5.750)	(-2.750)	(-2.520)
lnhhr	0.063	0.950***	0.281**	2.934***	-0.853	-1.905
	(0.860)	(3.370)	(2.290)	(4.040)	(-0.270)	(-0.050)
lnshr*lnhhr		-0.080***		-0.228***		0.092
		(-3.250)		(-3.690)		(0.030)
Intercept	Yes	Yes	Yes	Yes	Yes	Yes
Observations	248(31)	248(31)	155(31)	155(31)	62(31)	62(31)
R^2^	0.995	0.995	0.965	0.966	0.971	0.971

The t values are in parentheses below the coefficients; *, *, and *** represent significance at the 0.1, 0.05, and 0.01 levels, respectively

### Robustness test with dynamic panel model application

Resource endowment is the original capital for the development of medical services, and resource accumulation is an important link in achieving a stable improvement in the level of medical services. Considering the long training period for health human resources, there may be a certain lag in the level of medical services under its influence; therefore, a dynamic panel model is constructed. To further address the issue of endogeneity and verify the impact of variables, the system GMM method is used for estimation [44]. The system GMM method includes the “one-step method” and the “two-step method,” and for the sake of robustness, results from both methods are reported. In this context, the scale and hierarchical structure of health human resources and their interaction terms are considered endogenous variables, while the remaining variables are exogenous. The results, as shown in Table 8, indicate that the models pass both the Sargan test and the serial correlation test.

In Table 8, the second-order lagged terms of the dependent variable are all significantly positive, indicating that the historical level of medical services provides important support for the current level of medical services. All columns show that the scale coefficient and the structure coefficient are significantly positive at the 1% level, but the interaction terms are mostly significantly negative at the 1% level. This is basically consistent with the results mentioned above, confirming that both the scale and hierarchical structure of health human resources have a positive effect on the level of medical services. However, under the condition of scale, the impact of the hierarchical structure on the level of medical services becomes negative, indicating that an imbalance in the hierarchical structure has restrained the impact of the scale of health human resources on the level of medical services. This reflects the current urgent need to adjust the scale of health human resources and optimize the hierarchical structure. In the aforementioned “interactive mechanism,” only the unilateral positive effect of medical service efficiency on medical service equity remains significant, which supports the notion that equity in medical services is the “driving force” of the interactive mechanism. While short-term efficiency can ensure the balance of medical services, to sustain the vibrancy of the level of medical services, attention must be paid to and reliance placed on the equalization of medical services.

**Table 8:** System GMM estimation of the impact of health human resource scale and hierarchical structure on the level of medical services

	(1)	(2)	(3)	(4)
	lnhb		lnals	
	Two-step	One-step	Two-step	One-step
l2.lnhb	0.149***	0.205***		
	(3.060)	(2.980)		
l2.lnals			0.581***	0.461***
			(3.720)	(4.030)
lnshr	0.598***	0.584***	0.432***	0.344***
	(6.720)	(6.280)	(3.930)	(4.710)
lnhhr	1.307***	0.898***	1.085***	1.632***
	(4.830)	(3.120)	(2.860)	(5.780)
lnshr*lnhhr	-0.124***	-0.080***	-0.058	-0.127***
	(-3.950)	(-3.100)	(-1.270)	(-4.810)
lnals	-0.124**	-0.091		
	(-2.110)	(-1.640)		
lnhb			-0.088	-0.050
			(-1.520)	(-1.140)
Intercept	Yes	Yes	Yes	Yes
AR	0.141	0.323	0.027	0.000
Sargan test	0.000	0.000	0.007	0.007
Observations	341(31)	341(31)	341(31)	341(31)

The z values are in parentheses below the coefficients; *, **, and *** represent significance at the 0.1, 0.05, and 0.01 levels, respectively

### Robustness test with core explanatory variable substitution

Health personnel include medical technicians, administrative managers, support staff, and other technical personnel. To analyze the impact of different health personnel on the level of medical services, the variable for the scale of health human resources is replaced with the total number of health personnel. At the same time, the measurement method for the hierarchical structure variable is changed to the ratio of the sum of doctors and nurses to the total number of health personnel. Partial reproduction results are shown in Table 9.

Table 9 shows a strong consistency with the previous results, and the conclusions are essentially robust. Moreover, in columns (1) and (2), the coefficients for the hierarchical structure of health human resources and the interaction term change from insignificant to significant, indicating that, in addition to doctors and nurses, other types of health personnel may also be a channel to enhance the impact of health human resources on the level of medical services. In addition, due to some missing data in certain provinces in 2009, this study uses the Grey Prediction Model for supplementation. Therefore, after excluding the data of that year and repeating the empirical operations from the previous text, the results remain robust. Due to space limitations, this paper does not present these results.

**Table 9:** Partial empirical results after changing the explanatory variables

	(1)	(2)	(3)	(4)
	lnhs		lnals	
lnshr	0.730***	0.815***	0.201***	0.434***
	(12.970)	(11.540)	(4.810)	(7.770)
lnmsht	0.771**	-2.909	0.605***	-6.109***
	(2.080)	(-1.540)	(3.120)	(-5.350)
lnshr*lnmsht		0.324**		0.587***
		(1.980)		(5.950)
Intercept	Yes	Yes	Yes	Yes
Observations	403(31)	403(31)	403(31)	403(31)
R^2^	0.972	0.972	0.989	0.990

The t values are in parentheses below the coefficients; *, **, and *** represent significance at the 0.1, 0.05, and 0.01 levels, respectively; lnhps represents the total number of health personnel, and lnmhr represents the ratio of the sum of physicians and nurses to the total number of health personnel.

### Based on the channel test of different types of health personnel

Administrative staff, support staff, and other technical personnel, as important components of health human resources, play a central regulatory role in the operation of medical and health institutions. At the same time, the aforementioned research results indicate that other types of health personnel may also be a channel to enhance the impact of health human resources on the level of medical services. Therefore, this study draws on the approach of Acemoglu et al. [45] and conducts a channel test. The specific method is to introduce the total number of administrative staff, support staff, and other technical personnel, as well as the interaction terms of the above three with the total number of health personnel in the original model, and then observe the significance and coefficient changes of the original health human resources scale, hierarchical structure, and their interaction terms. If there is a significant decrease in the significance level or a change from significant to insignificant of the original interaction term, it is considered that this type of health personnel is the main regulatory effect channel. In addition, considering that health technical personnel are highly collinear with licensed (assistant) physicians and registered nurses, the impact of health technical personnel on the level of medical services is not analyzed separately in this study.

In Table 10, columns (2) and (8) show that after introducing different types of health personnel, the significance of the original interaction term decreases, indicating that administrative personnel are an important channel for regulating the impact of medical staff on the equity of medical services. It also reveals that other technical personnel are the key path for regulating the impact of medical staff on the efficiency of medical services. Administrative personnel can indirectly affect the equity of medical services through management decisions and policy implementation. Although other technical personnel do not directly participate in frontline clinical work, their technical work in health promotion, scientific research, and teaching can directly or indirectly alleviate the pressure on hospitals, thereby improving the efficiency and quality of medical services. The support staff’s impact on the level of medical services affected by medical personnel is not significantly different, which may be because the main work of support staff is concentrated in skilled operations and maintenance, logistical support services, and other aspects. Although essential for the daily operation of the hospital, their role in medical services may be more reflected in assisting the normal operation of the hospital rather than directly improving the level of medical services.

**Table 10:** Channel test based on different types of health personnel

	(1)	(2)	(3)	(4)	(5)	(6)	(7)	(8)
	lnopv				lnals			
lnmhr	0.624***	0.511***	0.707***	0.660***	0.187***	0.212***	0.176***	0.142***
	(9.730)	(6.950)	(9.260)	(9.540)	(4.070)	(3.970)	(3.190)	(2.870)
lnhhr	0.896***	1.179***	0.770**	0.662*	1.566***	1.506***	1.584***	1.865***
	(2.850)	(3.630)	(2.410)	(1.850)	(6.940)	(6.390)	(6.870)	(7.320)
lnmhr*lnhhr	-0.106***	-0.135***	-0.097***	-0.084**	-0.142***	-0.136***	-0.143***	-0.170***
	(-3.730)	(-4.540)	(-3.360)	(-2.570)	(-6.940)	(-6.310)	(-6.900)	(-7.300)
lnmgmt*lnhps		0.130***				-0.028		
		(3.050)				(-0.900)		
lnS&W*lnhps			-0.076**				0.011	
			(-1.980)				(0.390)	
lnot*lnhps				-0.038				0.048**
				(-1.380)				(2.460)
Intercept	Yes	Yes	Yes	Yes	Yes	Yes	Yes	Yes
Observations	403(31)	403(31)	403(31)	403(31)	403(31)	403(31)	403(31)	403(31)
R^2^	0.973	0.974	0.974	0.973	0.990	0.990	0.990	0.990

The t values are in parentheses below the coefficients; *, **, and *** represent significance at the 0.1, 0.05, and 0.01 levels, respectively; lnmgmt represents the natural logarithm of the number of managerial personnel, lnS&W represents the natural logarithm of the number of support and service workers, and lnot represents the natural logarithm of the number of other technical personnel

## Discussion

### The scale and hierarchical structure of health human resources have a significant impact on the enhancement of the level of medical services

The scale and hierarchical structure of health human resources are core elements affecting the level of medical services. This study found that the scale of health human resources significantly promotes medical service equity but exhibits an inhibitory effect on efficiency; meanwhile, the hierarchical structure of health human resources positively affects both the equity and efficiency of medical services. However, when the interactive effect of the scale and hierarchical structure of health human resources is included, it is observed that their interaction term promotes medical service equity but constrains the efficiency of medical services. Additionally, there is a bidirectional promotional relationship between the equity and efficiency of medical services, meaning that the equity of medical services can enhance the improvement of medical service efficiency, and vice versa. The reason behind this is that an expanded scale of health human resources implies a greater availability of medical professionals for allocation, thereby increasing the accessibility of medical services, while a rational hierarchical structure of health human resources contributes to the effective distribution of medical resources. The division of labor and collaboration among medical personnel at different levels can improve the overall efficiency of medical services. Therefore, the government should increase investment in medical education, steadily increase the stock of health talent through methods such as establishing more medical colleges, expanding enrollment scales, and increasing targeted training programs. At the same time, the rational allocation of health talent resources such as physicians, nurses, technicians, and pharmacists, and the creation of a reasonable ratio of health human resources, can help improve the efficiency of medical services and achieve the collaborative optimization of medical resources.

### Geographic divisions and the implementation of the “Outline” can intervene in the role of health human resources at the level of medical services

Through the examination of regional heterogeneity and temporal stages across the four major economic regions, it is revealed that both the economic regions and the periods before and after the implementation of the “Outline” have a significant impact on the role of the scale and hierarchical structure of health human resources in the level of medical services. In the western and northeastern regions, the hierarchical structure of health human resources plays a particularly significant role in promoting the level of medical services, while the effect is less pronounced in other areas. This indicates that there is ample room for improvement in the level of medical services in the western and northeastern regions, while the central and eastern regions suffer from an overflow of high-quality resources, hindering further development. In the temporal heterogeneity test taking the implementation of the “Outline” as a breakpoint, there is a significant difference in the impact of health human resources on the level of medical services before and after 2016. After the implementation of the Outline, the role of health human resources in promoting the level of medical services has become more pronounced, demonstrating that national regulation is crucial in the development of health human resources. Therefore, when optimizing the allocation of health human resources, the intervention effect of health policy in optimizing the allocation of health human resources and improving the level of medical services should be given importance. The characteristics and needs of different economic regions should be fully considered. In economically developed regions such as the east and central regions, it is necessary to remain highly vigilant against the expansion of the “top,” adhering to high standards of talent positioning and quality bottom lines. In contrast, the western and northeastern regions still need to focus on consolidating the “foundation” as their primary work goal, continuing to increase the stock of health human resources. In addition, it is necessary to actively promote the output of talent from “leading” regions and the introduction of talent in “lagging” regions. At the same time, continuous monitoring and evaluation of the implementation effects of policy guidelines, adjusting the ratio of health human resources, ensuring a balanced structure of human resources, and serving the high-quality development of medical services are essential.

### The conclusions remain robust with the implementation of core explanatory variable substitution and the use of dynamic GMM model adjustments

In the robustness test, by substituting the core explanatory variables, it was found that the model remains stable, further verifying the reliability and robustness of the research results. Moreover, after employing the dynamic GMM model, the second-order lag of the medical service level is significant, reflecting the substantial impact of the accumulation of medical service resources in the previous period on future medical services, and indicating a stronger lag effect on medical service efficiency compared to the equity of medical services. The channel test proves that managerial personnel and other technical personnel play a key role in regulating the impact of medical staff on the medical service level. Over the past 30 years, the significant changes in China’s disease spectrum, especially the increase in the burden of chronic non-communicable diseases, demand a shift in the medical service model from “disease-centered” to “patient-centered” [46]. To this end, it is necessary to build multidisciplinary teams (MDT), integrating health talents from different professional backgrounds to optimize the allocation of medical resources and service processes, thereby enhancing the overall level of medical services. In addition, in the construction of medical services, a combination of long-term and short-term strategies is needed. In the short term, attention should be paid to the basic investment in the construction of medical services to lay a solid foundation for the sustainable development of medical services. Long-term planning should consider various factors such as regional economic development, government regulatory capacity, social support level, and people’s health needs. It is necessary to formulate long-term plans for the development of health human resources and implement specific and feasible short-term plans to adapt to the cyclical development trend of medical services and establish a virtuous cycle in the medical service system.

## Conclusion

In China’s four major economic regions, the scale and hierarchical structure of health human resources can have a positive impact on the equity and efficiency of medical services, and geographical location and policy intervention are the two main factors affecting this impact. Therefore, when optimizing the allocation of health human resources and improving the level of medical services, it is necessary to consider the geographical location of each region comprehensively, actively and effectively implement relevant policy measures, and reasonably regulate the hierarchical structure of health human resources, rather than simply increasing the scale of health human resources. This study conducts macro-level research based on panel data from China’s four major economic regions, lacking in-depth analysis with hospitals or medical and health institutions as research subjects; follow-up studies can supplement and verify through the collection of micro-level databases.

## Data Availability

No datasets were generated or analysed during the current study.

## Abbreviations

WHO: World Health Organization
CNY: Chinese yuan
GDP: Gross Domestic Product
GMM: Generalized Method of Moments
COVID: Corona Virus Disease
MDT: Multi-Disciplinary Treatment
